# The immune microenvironment: a key regulator of ovarian function during ovarian aging

**DOI:** 10.3389/fimmu.2026.1857242

**Published:** 2026-06-15

**Authors:** Tingting Lin, Wei Zhao, Yaoqing Xie, Jianyong Xu, Su Liu, Ling Hong, Jianwen Mao, Chunyu Huang

**Affiliations:** 1 College of Basic Medicine, Guangdong Pharmaceutical University, Guangzhou, China; 2 Clinical Research Center for Reproductive Medicine, Shenzhen Zhongshan Obstetrics and Gynecology Hospital (formerly Shenzhen Zhongshan Urology Hospital), Shenzhen, China; 3 Shenzhen Zhongshan Institute for Reproduction and Genetics, Shenzhen, China; 4 Guangdong Provincial Engineering Technology Research Center for Peri-implantation Reproductive Immunology, Shenzhen, China

**Keywords:** fertility preservation, immune-targeted therapy, ovarian aging, ovarian immune microenvironment, premature ovarian insufficiency

## Abstract

The ovary is a highly dynamic organ characterized by intense biosynthetic activity, cyclical tissue remodeling, and elevated metabolic flux. Within this demanding milieu, precise immune regulation is paramount: tightly coordinated tissue remodeling by innate immunity and vigilant immune surveillance by the adaptive system are critical for maintaining ovarian homeostasis. Mounting evidence identifies dysregulation of the local ovarian immune microenvironment as a core driver of functional decline. This review synthesizes current knowledge on how the ovarian immune microenvironment, in concert with endocrine signals, orchestrates key phases of oocyte development and follicular maturation. We then delineate the immune signatures underpinning physiological ovarian aging and pathological premature ovarian insufficiency, highlighting the mechanistic continuum between these conditions. Finally, we systematically review emerging immunomodulatory interventions targeting the ovarian immune microenvironment, including strategies that restore Treg/Th17 balance, modulate macrophage polarization, and employ mesenchymal stem cells or platelet-rich plasma to remodel the immune niche. By integrating these mechanistic and therapeutic insights, this review aims to establish a coherent theoretical framework and future research directions for developing novel ovarian-protective interventions designed to improve reproductive outcomes and long-term health in aging women and those with POI.

## 1 Introduction

The ovaries play a central role in the female reproductive and endocrine systems, primarily responsible for oocyte development, sex hormone secretion, and maintaining overall health. Throughout the lifespan, ovarian function follows a tightly regulated pathway: beginning with the establishment of the primordial follicle pool during fetal development, reaching peak reproductive capacity in early adults, and subsequently experiencing a gradual decline in both quantity and quality of follicles with advancing age. This age-related transformation fundamentally influences female fertility and metabolic health.

After the mid-30s, women experience a significant acceleration in follicle depletion, marking a critical transition in ovarian reserve and function. Clinically, this change manifests primarily through several factors: declining Anti-Müllerian Hormone (AMH) levels, reduced antral follicle count, increased chromosomal risks associated with aneuploid oocytes, and a corresponding decrease in the success rates of assisted reproductive technologies (ART) (1, 2). These physiological changes lead to a decline in natural fertility and increased reproductive difficulties. Under the global trend of delayed childbearing, women aged 35 and older have become the primary group seeking infertility treatment. Compared to younger women, they face significantly higher risks of infertility and relatively poorer outcomes with ART (3, 4). This gradual decline culminates during menopause, typically around ages 50–51, marking the end of reproductive capacity and the onset of systemic estrogen deficiency effects on skeletal, cardiovascular, and neurocognitive health. These age-related transitions collectively underscore the intrinsic link between ovarian aging, reproductive potential, and women’s long-term health. It is noteworthy that, in addition to the common age-related functional decline, some women may experience a pathological, rapid loss of function known as premature ovarian insufficiency (POI). POI is strictly defined as ovarian failure occurring before the age of 40. Although its clinical manifestations (e.g., amenorrhea with elevated gonadotropin levels) may resemble postmenopausal states, its onset occurs significantly earlier than the physiologically normal timeline. The global prevalence of POI is approximately 3.5% (5), this condition directly causes infertility, requiring patients to rely on specialized ART such as oocyte donation. Additionally, chronic sex hormone deficiency significantly increases the risk of osteoporosis, cardiovascular disease, and cognitive impairment. Therefore, elucidating the molecular mechanisms underlying ovarian aging and POI development is urgently needed.

The etiology of POI is multifactorial, encompassing genetic defects, iatrogenic damage (e.g., chemotherapy or ovarian surgery), exposure to environmental or toxic substances, and autoimmune diseases. However, over 70% of cases remain classified as idiopathic (5). Although these classifications summarize the potential triggers of POI, they struggle to explain the significant heterogeneity in the onset timing, progression rate, and clinical outcomes of the condition. Even when exposed to similar types and degrees of gonadal toxic damage, the trajectories of ovarian function decline can vary greatly among individuals. This suggests the potential existence of common regulatory mechanisms influencing the repair capacity or susceptibility of the ovaries. This also suggests that POI cannot be adequately understood through a single etiological factor alone. We must explore relevant regulatory mechanisms at the systemic and tissue levels.

In recent years, the ovarian microenvironment has emerged as a dynamic regulatory core integrating endocrine, metabolic, vascular, stromal, and immune signaling. Within this microenvironment, the immune microenvironment constitutes a critical yet under-explored central component. Immune cells such as macrophages, regulatory T cells (Tregs), dendritic cells (DCs), innate lymphoid cells, and B cells interact with granulosa cells and stromal cells through cytokines, chemokines, and metabolic mediators, collectively maintaining follicular homeostasis, tissue remodeling, and immune tolerance (6). Once this finely tuned immune balance is disrupted, it can lead to chronic low-grade inflammation, disruption of immune tolerance mechanisms, and abnormal repair processes, thereby accelerating follicular atresia, stromal fibrosis, and endocrine dysfunction.

**Figure 1:**
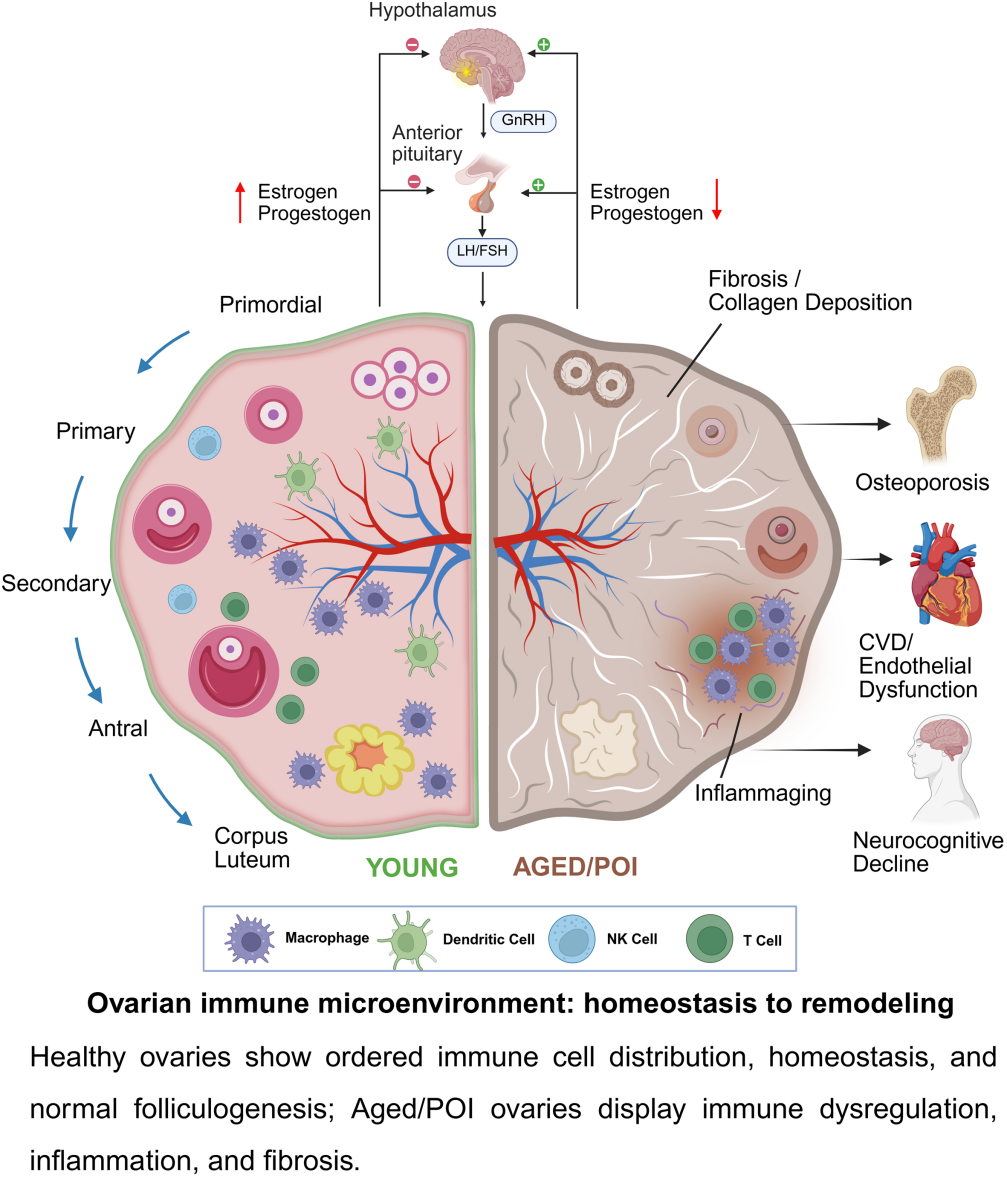
Ovarian immune microenvironment: homeostasis to remodeling. Healthy ovaries show ordered immune cell distribution, homeostasis, and normal folliculogenesis; Aged/POI ovaries display immune dysregulation, inflammation, and fibrosis.

Importantly, immune dysregulation may represent a mechanistic bridge connecting physiological ovarian aging and POI. Physiological ovarian aging is accompanied by a chronic low-grade inflammatory state characterized by macrophage reprogramming, increased production of proinflammatory cytokines (e.g., IL-6 and TNF-α), and impaired immune tolerance (6). Similar immune alterations have been observed in POI, including elevated inflammatory cytokines, altered macrophage polarization, and disrupted Treg/Th17 balance. These changes correlate with diminished ovarian reserve and follicular loss (7, 8). These similarities suggest that alterations in the immune microenvironment are not merely secondary phenomena following ovarian dysfunction, but rather a key mechanism underlying both physiological ovarian aging and idiopathic POI. Increasingly, it is recognized that elucidating physiological ovarian aging may provide crucial insights into the pathogenesis of idiopathic POI. Although physiological ovarian aging and POI differ in onset timing and clinical progression, both manifest as accelerated follicular depletion, stromal remodeling, and endocrine dysfunction. In susceptible individuals, genetic predisposition, metabolic stress, or environmental damage may prematurely trigger aging-related immune alterations (e.g., chronic inflammation, impaired Treg function, and diminished tissue repair capacity). By extension, idiopathic POI may be regarded as the premature activation or dysregulation of aging-related immune pathways in specific individuals, rather than a wholly distinct disease entity (**Figure 1**).

Despite the preceding insights, a systematic understanding of immune cell heterogeneity, spatiotemporal dynamics, and their interactions with stromal/germ cells remains elusive. This knowledge gap hinders the identification of early biomarkers, blurs the distinction between physiological aging and pathological immune dysregulation, and constrains the development of targeted therapies aimed at preserving ovarian function. We propose that immune microenvironment remodeling may represent a key associative hub between physiological ovarian aging and POI. This framework provides a theoretical foundation for mechanism-based diagnosis, risk stratification, and immunomodulatory interventions, ultimately enabling ovarian function preservation and extended reproductive lifespan.

## 2 The ovarian immune microenvironment in physiological function

The ovary is a structurally heterogeneous organ composed of the cortex, medulla, and surface epithelium, each forming a distinct microenvironment to support follicular development and endocrine function (9). The cortex contains follicles at various stages of development, embedded within a matrix rich in fibroblasts, extracellular matrix components, and a network of blood vessels (10). The medulla is characterized by a dense network of blood vessels and connective tissue, serving as a vital pathway for immune cell migration and hormone exchange.

Within this organizational structure, the follicular unit functions as a specialized microenvironmental niche where the oocyte, granulosa cells, theca cells, stromal cells, endothelial cells, and extracellular matrix components interact through paracrine signaling and metabolic signaling (11, 12). This spatial architecture not only supports follicular development but also governs the localization, activation status, and functional interactions of immune cells. The vascular network and stromal scaffold regulate immune cell recruitment, while extracellular matrix remodeling influences immune cell retention and cytokine diffusion. Consequently, the ovarian immune microenvironment is inextricably linked to its structural environment, with tissue architecture serving as a key determinant of immune regulation.

**Figure 2:**
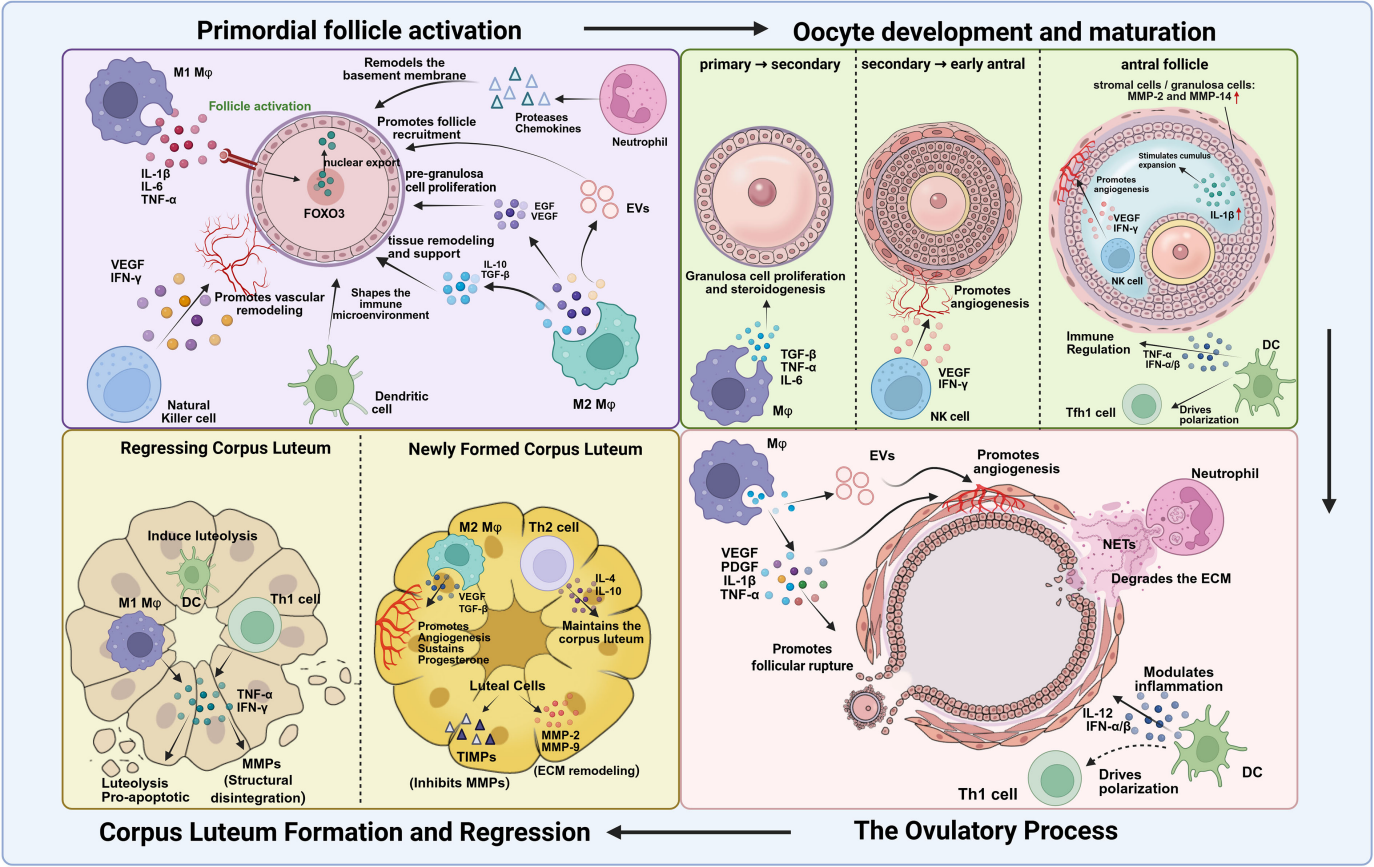
Immune microenvironment regulates ovarian follicle development. Immune cells and their mediators (cytokines, chemokines, proteases) coordinate primordial follicle activation, oocyte maturation, ovulation, and corpus luteum turnover. Immune signaling drives follicle recruitment, vascular remodeling, and granulosa cell expansion during folliculogenesis; mediates extracellular matrix breakdown and angiogenesis for ovulation; and regulates luteal formation, progesterone sustainment, and structural regression, thereby sustaining ovarian cyclicity.

Building upon this structural framework, a dynamic immune microenvironment exists within the ovary, spanning its anatomical regions and developmental stages. Tissue-resident macrophages, DCs, natural killer (NK) cells, T/B lymphocytes, mast cells, and neutrophils are widely distributed throughout the ovarian cortex, medulla, stroma, follicular fluid, and surface epithelium. Overall, within the follicular fluid, immune cells can constitute up to 50% of non-germ cells (13). These immune cells and the cytokines they secrete not only perform host defense functions but also actively participate in follicular development, ovulation, maintenance of corpus luteum function, and regulation of ovarian homeostasis (14–20) (**Figure 2**).

### 2.1 Activation of primordial follicles

The study confirmed that the proportion of CD11c^+^ M1-type macrophages subpopulations significantly increased around developing follicles, while the proportion of CD206^+^ M2-type macrophages subpopulations showed no significant change. Furthermore, depletion of CD11c^+^ M1 subpopulations resulted in follicular damage and hemorrhage, whereas depletion of CD206^+^ M2 subpopulations did not produce this effect (21). Therefore, during the primordial follicle activation phase, macrophages exhibit marked heterogeneity and spatiotemporal plasticity within the ovarian cortex. Promyelocytic-type M1 macrophages transiently release IL-1β, IL-6, and TNF-α. These cytokines synergistically activate the PI3K/Akt/mTOR and JAK/STAT3 signaling pathways in oocytes and pre-granulosa cells, thereby reducing nuclear retention of FOXO3 and triggering cell cycle initiation (22–24). In parallel, macrophage-derived growth factors, including EGF, IGF, HGF, and VEGF, stimulate pre-granulosa cell division and angiogenesis, providing both structural and metabolic support for nascent follicles (25). Macrophage phenotypes exhibit a continuous spectrum distribution. Among them, the M2 subpopulation secretes IL-10 and TGF-β, suppressing excessive inflammatory responses, remodeling the extracellular matrix, and maintaining vascular stability, thereby collectively safeguarding the structural integrity of follicles (26–28). Notably, extracellular vesicles (EVs) derived from macrophages can transmit microRNAs, proteins, and lipids between cells. Research confirms that EVs originating from reparative macrophages enhance PI3K/Akt signaling pathway activity, mitigate inflammatory aging, and promote the recruitment of primordial follicles in aged ovaries. This offers a potential therapeutic strategy for delaying follicular reserve depletion (29, 30).

In addition to macrophages, DCs promote follicular expansion, oocyte release, and corpus luteum formation through gonadotropin-dependent recruitment and upregulation of key ovulation genes. These CD11c-positive, F4/80-negative DCs also possess anti-inflammatory capabilities, limiting ovulation-associated inflammatory responses (31). Meanwhile, innate lymphoid cells such as NK cells regulate local angiogenesis and tissue remodeling by releasing VEGF and IFN-γ (32). Neutrophils, as the first wave of cells responding to acute ovarian stress, release proteases and chemokines to remodel the basement membrane, thereby decreasing the threshold for follicular awakening (33, 34). Collectively, primordial follicle activation emerges not from a single pathway but from the coordinated interactions among macrophage subtypes, other innate and adaptive immune cells, and their soluble factors and EVs, which jointly dictate the amplitude, frequency, and reversibility of follicle awakening.

### 2.2 Oocyte development and maturation

As oocytes sequentially develop through the primary and secondary stages, particularly during the follicular phase, immune cells and their secreted mediators continue to exert critical and stage-specific roles throughout oocyte maturation.

Macrophages constitute the predominant white blood cell population within the follicular membrane layer and ovarian stroma. Growing evidence indicates that these cells exert complex regulatory functions through multiple mechanisms. As a major source of local cytokines and growth factors, macrophages secrete paracrine mediators such as TGF-β, TNF-α, and IL-6, promoting granulosa cell expansion, angiogenesis, and steroid hormone synthesis. Macrophages also produce chemokines and other growth factors, participating in tissue remodeling and enhancing oocyte developmental potential through phagocytosis, matrix degradation, and clearance of exogenous antigens (23). Beyond their secretory function, macrophages are crucial for maintaining ovarian vascular integrity (28). In a conditional macrophage knockout mouse model expressing CD11b-diphtheria toxin receptor (DTR), specific depletion of ovarian macrophages by diphtheria toxin (DT) induced progressive ovarian hemorrhage in both corpus luteum and stromal regions. This was accompanied by reduced CD31+ endothelial cell numbers, increased red blood cell accumulation, and accelerated follicular atresia (28). These findings suggest that macrophages play an indispensable role in maintaining endothelial cell survival and vascular stability, a function potentially linked to their periodic distribution pattern within ovarian tissue. With advancing age, macrophages undergo significant phenotypic and functional alterations accompanied by dysregulated signaling pathways. Single-cell RNA sequencing and flow cytometry analysis of ovaries from young (3 months) and aged (14–17 months) mice revealed marked disruption of the ANNEXIN and TGF-β signaling pathways in aged ovarian myeloid cells (35). In summary, these studies confirm that macrophages influence ovarian function through cytokine secretion, maintenance of vascular homeostasis, and regulation of age- and stage-specific signaling pathways, thereby coordinating granulosa cell expansion, angiogenesis, steroid hormone synthesis, and oocyte developmental potential.

NK cells, particularly the CD56^+^CD16^-^ proangiogenic subset, accumulate in follicular fluid during the follicular sinus phase and preovulatory phase. They secrete VEGF and IFN-γ, thereby promoting angiogenesis and creating a suitable microenvironment for oocyte development. Conversely, the proportion of cytotoxic CD16^+^ NK cell subpopulations decreases, and this shift correlates positively with normal follicular maturation (36, 37).

In addition to macrophages and NK cells, DCs infiltrate the follicular membrane layer during the sinusoidal follicular phase, serving as antigen-presenting cells that bridge innate and adaptive immunity. Activated ovarian DCs secrete TNF-α and type I interferon, indirectly regulating the activity of granulosa and theca cells by shaping the local follicular microenvironment. Mechanistically, these DCs can drive local T cell differentiation toward a type 1 follicular helper T cell (Tfh1) phenotype via a CD40 dependent pathway during inflammation. Specifically, a subset of DCs expressing high levels of CD40 and low levels of ICOS-L interacts with naive CD4^+^ T cells, inducing co-expression of BCL6 and TBET, upregulating PD-1, CXCR5, and ICOS molecules, and secreting large amounts of interleukin-21 (IL-21) and IFN-γ. This Tfh1 polarization may promote local antibody-mediated immune responses, enhance follicular immune surveillance, and potentially influence oocyte quality and follicular development by regulating the cytokine microenvironment (38–40). Experimental depletion of ovarian DCs in animal models impairs lymphangiogenesis, disrupts cumulus-oocyte complex expansion and oocyte release, and compromises functional corpus luteum formation, suggesting that DCs play a crucial supportive role in the immune microenvironment surrounding ovulation (41).

The latest immunological profiling of follicular fluid further reveals significant differences in its immune microenvironment compared to peripheral blood. Follicular fluid exhibits elevated levels of proangiogenic cytokines (e.g.,VEGF) (42), enriched macrophages, Th22 cells, tissue-resident cDC2 cell (37, 43), and reduced classical monocyte and CD8^+^ T cell frequencies (37, 42). Matrix metalloproteinases (MMPs), including MMP-2 and MMP-14, are highly expressed in ovarian stromal cells and granulosa cells near the tertiary follicles. They mediate matrix remodeling and dominant follicle selection by degrading the extracellular matrix (44). Compared to plasma, follicular fluid contains higher concentrations of cytokines such as VEGF and EGF. Research confirms that these factors promote follicular development by increasing capillary density and follicular numbers (45). The VEGF/VEGFR-2 signaling pathway within the ovary is essential for gonadotropin-dependent angiogenesis and the final maturation of follicles (46). IL-1β exerts bidirectional regulatory effects. At physiological concentrations, it promotes cumulus expansion and oocyte maturation, whereas sustained elevation of its concentration inhibits oocyte maturation and reduces oocyte quality (47). Members of the EGF family promote oocyte maturation by triggering granulosa vesicle breakdown (GVBD), first polar body expulsion, and expansion of the cumulus-oocyte complex (47).

In summary, macrophages, NK cells, DCs, MMPs, and paracrine cytokines synergistically regulate matrix remodeling, angiogenesis, granulosa cell activity, and oocyte maturation. These signals interact with adaptive immune modulators in the follicular fluid to establish a supportive microenvironment, which is crucial for successful oocyte maturation, ovulation, and subsequent reproductive capacity.

### 2.3 Immunomodulation of the ovulatory process

Ovulation is a tightly regulated physiological process characterized by acute inflammatory features. During this process, gonadotropin signaling integrates innate and adaptive immune mechanisms, triggering local proteolytic, vascular, and endocrine changes that provide the necessary conditions for follicular rupture and oocyte release. The preovulatory luteinizing hormone (LH) surge induces granulosa and theca cells to express chemokines (e.g., MCP-1/CCL2, CXCL8/IL-8) and proinflammatory cytokines (primarily IL-1β and IL-6) (48, 49), thereby recruiting neutrophils, monocytes/macrophages, and DCs to infiltrate the follicular wall. These recruited leukocytes amplify the local inflammatory microenvironment by inducing cyclooxygenase-2 (COX-2) to enhance PGE2/PGF2α synthesis and increasing local protease activity (e.g., MMPs, plasminogen activators). Collectively, they degrade the follicular extracellular matrix, ultimately promoting follicular rupture (6, 49). Animal models and *in vitro* ovarian perfusion studies indicate that IL-1β is a key regulatory molecule. In rodent and rabbit *in vitro* ovarian systems, IL-1β alone can induce ovulation. IL-1β potentiates the ovulation-inducing effects of LH/human chorionic gonadotropin (HCG) and promotes ovarian prostaglandin and progesterone release, with these effects at least partially dependent on the COX-2 pathway; conversely, blocking IL-1 signaling reduces ovulation efficiency (50–52). Recent studies have revealed that activation of the NLRP3 inflammasome and caspase-1 within the ovaries occurs upstream of IL-1β maturation, linking pattern-related or danger-related signals to amplified IL-1β production and ovulation-associated inflammation. This mechanism provides a molecular node for metabolic or aseptic stress to regulate ovulatory capacity (53).

Neutrophils are early effector cells in preovulatory follicles. Recruited by chemotactic factors such as IL-8/CXCL8 (54), they release proteolytic enzymes and MMPs and can form neutrophil extracellular traps (NETs), thereby participating in matrix remodeling and the formation of local cytokine concentration gradients (33). Animal model studies demonstrate that experimentally depleting neutrophils or inhibiting neutrophil recruitment attenuates the matrix remodeling process and impairs ovulation, indicating that neutrophils actively participate in the mechanical and enzymatic processes associated with follicular rupture (55).

Macrophages exist in two forms: tissue-resident and recruited, exhibiting a dynamic M1/M2-like polarization phenotype. These cells secrete various proteases, growth factors (e.g., VEGF, PDGF), and cytokines (IL-1β and TNF-α), thereby promoting local angiogenesis, increasing vascular permeability, and mediating tissue remodeling. These processes are critical for follicular rupture and subsequent corpus luteum formation (29, 56). Beyond cell recruitment, studies indicate that EVs secreted by immune cells and their carried microRNAs (miRNAs), particularly those derived from macrophages, can regulate granulosa cell signaling pathways. They activate pathways associated with angiogenesis and cell expansion, and exert fine-tuned control over inflammatory responses during the pre- and post-ovulation phases (29). This extracellular vesicle-mediated intercellular communication constitutes an additional regulatory layer that influences ovulation efficiency and the quality of released oocytes.

Under gonadotropin stimulation, DCs are recruited to the ovaries, where they promote ovulation by upregulating key ovulation-related genes. This process facilitates the expansion of the cumulus-oocyte complex, oocyte release, and corpus luteum formation. Concurrently, DCs exert anti-inflammatory effects, thereby limiting inflammation-related tissue damage (41).

Recent studies underscore that the inflammatory response mediating ovulation must be transient and spatially confined. Excessive, persistent, or dysregulated inflammatory responses, including chronic NLRP3 inflammasome activation, elevated IL-1β levels, and abnormal neutrophil or NET function, have been associated with diminished oocyte quality and ovulatory dysfunction (57, 58). In summary, these findings support a more refined regulatory model. Under LH induction, localized innate immune activation, centered on IL-1β maturation, matrix remodeling mediated by neutrophils and macrophages, regulated EV signaling, and tightly controlled angiogenesis, establishes a controlled, self-limiting inflammatory microenvironment. This process mediates follicular rupture and lays the foundation for luteinization and early corpus luteum formation (59, 60).

### 2.4 Immunomodulation of corpus luteum formation and degeneration

Following oocyte release, the remaining follicular structure undergoes a rapid transformation into the corpus luteum, a process akin to wound healing and *de novo* organogenesis, followed by its eventual demise (luteolysis). Immune cells contribute to corpus luteum formation, also known as luteogenesis. The initial hemorrhagic wound is infiltrated by M2 macrophages and Tregs. These cells suppress detrimental inflammation, clear erythrocyte debris via phagocytosis, and secrete copious amounts of VEGF, PDGF, and TGF-β, which promote rapid neovascularization characteristic of early luteal tissue and ensure adequate delivery of cholesterol substrates and oxygen for progesterone synthesis (61, 62). Depletion of macrophages in murine models results in impaired corpus luteum vascularization, decreased luteal progesterone production, and compromised implantation, highlighting their indispensable role in luteal formation (61). In parallel, MMPs derived from leukocytes and luteal cells, including MMP-2 and MMP-9, are transiently upregulated during early luteal development, mediating extracellular matrix remodeling required for luteal expansion. Their activity is balanced by tissue inhibitors of metalloproteinases (TIMPs) secreted by luteal cells, thereby preventing excessive tissue breakdown (63, 64).

As the CL transitions into the regression phase in the absence of pregnancy, the immune landscape undergoes profound changes. Macrophages polarize toward a pro-inflammatory M1 phenotype and secrete cytokines such as TNF-α and IFN-γ, which induce apoptosis of luteal steroidogenic cells and promote functional luteolysis (6, 23). These cytokines also stimulate the production of MMPs, accelerating ECM degradation and structural collapse of the CL (65). Endothelial cell loss and vascular regression, driven in part by immune-mediated angiostatic signals, are critical features of luteolysis and contribute to decreased progesterone production (66). Macrophages then phagocytose the apoptotic debris, and fibroblasts deposit collagen, forming the avascular scar known as the corpus albicans. In addition to macrophages, T lymphocytes exert stage-dependent effects: a Th2-dominant cytokine profile characterized by IL-4 and IL-10 supports luteal maintenance, whereas a shift toward Th1 cytokines enhances regression (67). DCs and other antigen-presenting cells are also present within the CL, where they contribute to local immunosurveillance and resolution of inflammation, although their precise functions remain less defined compared to macrophages (41). Recent studies highlight the role of immune-mediated clearance of senescent luteal cells, positioning the immune system as a key executor of ovarian tissue homeostasis and cyclic renewal.

In summary, existing research evidence indicates that immune cells play a central role in both the formation and regression of the corpus luteum. Macrophages serve as indispensable regulators of angiogenesis, extracellular matrix remodeling, and luteal steroid hormone production, with shifts in their polarization states precisely orchestrating the transition from luteal maintenance to regression. T cells, DCs, and other immune subpopulations further refine this immune-endocrine equilibrium, creating a highly dynamic microenvironment that ultimately determines the fate of the corpus luteum.

## 3 Immune microenvironment alterations in ovarian aging and POI

Taken together, immune cells within the ovaries not only perform host defense functions but also participate in regulating follicular development and tissue remodeling. Excessive pro-inflammatory activity or abnormal autoantibody production disrupts this network, closely correlating with ovarian aging and pathological ovarian insufficiency. Therefore, systematically elucidating the dynamic changes in the immune microenvironment during physiological ovarian aging and revealing its dysregulation in POI is crucial for deepening our understanding of the common immunological mechanisms underlying ovarian decline and identifying potential immunotherapeutic targets (**Tables 1**, **2**).

**Table 1:** Summary of key studies investigating natural aging.

First author (year)	Species	Groups	Samples	Key findings	Technique
Jin et al., 2025 (69)	Human	Young: 23–29 years Aged: 49–54 years	Ovary	Immune Cells (CDKN1A^+^) ↑ CXCR4, IL6ST, FGF7, PDGF ↑	scRNA-seq
Wu et al., 2024 (70)	Human	Young:18–28 years Middle-aged:36–39 years Aged:47–49 years	Ovary	TNF-α, CXCL8 ↑ IL-1α, IL-1β, IL-6 ↑	scRNA-seq
Zhou et al., 2024 (68)	Human	Young <33 years Middle-aged >37 years	ovarian cortex	Tissue-resident Macrophages (C1QC^+^, SPP1^+^, HLA-DQA1^+^) ↓ Monocyte-Derived Macrophages (VCAN^+^) ↑	scRNA-seq IHC
				Effector T cells (FCGR3A+, FGFBP2+,GZMB+,GNLY+) ↓	scRNA-seq
				Total T cells(CD3+)↑	IF
Xu et al., 2021 (71)	Human	Young:31–34 years Aged:50–62 years	Ovary	Macrophages(CD14^+^/CD74^+^)↓ NK Cells (IL32^+^/CCL5^+^) ↓	scRNA-seq
Converse et al., 2025 (74)	Non-Human Primate and Mouse	6–12 Weeks vs. 9 Months vs. 12 Months vs. 15 Months	Ovary	Multinucleated Giant Cells(GPNMB^+^) ↑	Bulk RNA-seq
Isola et al., 2024 (72)	Mouse	3 Months vs. 9 Months	Ovary	B cells (CD19^+^), T cells (CD90^+^) ↑ Monocyte↑	scRNA-seq
Shen et al., 2024 (73)	Mouse	3 Months vs. 6 Months vs. 9 Months	Ovary	TNF-α, IL-18, IL-10↑	RT-PCR

**Table 2:** Summary of key studies investigating POI.

First author (year)	Species	Groups	Samples	Key findings	Technique
Tang et al., 2024 (109)	POI Patients	25 IU/L<FSH ≤ 40 IU/L	Peripheral Blood Follicular Fluid	IL-33↑	ELISA
Nouri et al., 2024 (77)	POI Patients	18 years <Age <40 years Amenorrhea ≥4 months FSH >25 IU/L (twice)	Peripheral Blood	Th17 Cells (IL17^+^, CD4^+^) ↑	FCM
				IL-17, IL-21, IL-23 ↑	qRT-PCR ELISA
Jiao et al., 2021 (76)	POI Patients	Age <40 years Amenorrhea ≥4 months FSH >25 IU/L (twice)	Peripheral Blood Follicular Fluid	TH1 Cells (CD4+, IFN-γ+) ↑ Treg Cells ( CD4+, CD25+, Foxp3+)↓	FCM
				IFN‐γ, TNF‐α↑ TGF-β1↓	ELISA
Kobayashi et al., 2019 (7)	POI Patients	Age <40 years Amenorrhea ≥4 months FSH >25 IU/L (twice)	Peripheral Blood	CD4 + T Cells ↑ Treg Cells (CD4^+^, Foxp3^+^) ↓	FCM
Yang et al., 2024 (80)	Mouse Model	CTX+BUS-POI	Ovary	M1 Macrophage (CD86^+^, INOS^+^) ↑ M2 Macrophage (CD206^+^, ARG-1^+^)↓ IL-6, TNF-α ↑	qRT-PCR WB
Ren et al., 2024 (81)	Mouse Model	CTX-POI	Ovary	M1 macrophage (CD86^+^) ↑	qRT-PCR WB
Deng et al., 2024 (78)	Mouse Model	pZP3-POI	Ovary	Th17 cells↑ Tregs↓	ELISA

### 3.1 Ovarian aging

In studies examining human ovarian aging, Zhou et al. performed single-cell RNA sequencing on the ovarian cortex from women ranging from reproductive age to middle age. Results revealed that with advancing age, expression of pyroptosis-related genes (e.g., GSDMD, CASP1) significantly increased within macrophage clusters. Concurrently, the macrophage phenotype shifted from tissue-resident to monocyte-derived inflammatory types. In a mouse model, GSDMD knockout partially alleviated stromal cell senescence, suggesting that macrophage pyroptosis directly contributes to pathological processes associated with ovarian aging (68). Based on the aforementioned findings regarding cellular inflammation and pyroptosis, Jin et al. employed multi-omics sequencing of monocytes to further investigate the functional remodeling of ovarian immune cells during aging. Although the proportion of immune cells remained relatively stable, the signaling capacity of aged immune cells was significantly impaired, particularly in receiving extracellular signals. This was accompanied by abnormal activation of aging-related pathways, manifested as an increased proportion of p21-positive cells and enhanced activation of the HIF-1 hypoxia signaling pathway. These findings indicate that ovarian immune aging is characterized by functional “signaling failure” and dysfunction, potentially associated with microenvironmental hypoxia (69). Beyond cellular dysfunction and hypoxia, Wu et al. further provided evidence for chronic low-grade inflammation in the aging ovarian immune microenvironment. Through single-cell RNA sequencing, the team discovered elevated scores in senescence signaling pathways, enhanced activation of the NF-κB pathway, and upregulation of proinflammatory factors such as IL-1β and IL-6 in aging ovarian immune cells. Collectively, these factors form a proinflammatory microenvironment that accelerates ovarian aging (70). Xu et al. further elucidated the dual alterations in both quantity and function of immune cells in aged ovaries through single-cell multi-omics analysis, thereby refining the aforementioned findings. Macrophages and NK cells were significantly reduced in aged ovaries. At the transcriptional level, abnormalities in binding and activation were observed in transcription factors associated with NK cell clonal expansion and memory formation, as well as in STAT6, which regulates M2 macrophage polarization. Collectively, aging impairs multiple core macrophage functions, disrupts macrophage-dependent immune regulation, and further exacerbates ovarian immune aging (71).

Beyond human single-cell transcriptional analyses, mouse studies have expanded our understanding of ovarian immune aging by exploring lymphocyte changes, macrophage functional deficits, and unique aged-related cell populations. For instance, regarding lymphocytes, Isola et al.’s mouse study revealed that the total number of immune cells in the ovaries doubled with age, with the most significant relative expansion occurring in lymphocyte subpopulations. This suggests that during the aging process, the presence and proportion of adaptive immune cells within the ovarian microenvironment undergo systematic alterations (72). At the macrophage level, Zhang et al. analyzed ovarian myeloid cells in mice and found that although the absolute number of macrophages did not necessarily increase significantly, aging led to elevated expression of MHC II molecules and proinflammatory cytokine genes such as IL1β and TNF-α within macrophage subpopulations (35). Shen et al. demonstrated that protein levels of macrophage scavenger receptor markers (CD36, CD68, CD204) in ovarian lysates from aged mice decreased by approximately 30%–40%, and that phagocytic capacity of isolated aged ovarian macrophages was significantly impaired compared to younger controls (73). Other morphological evidence indicates that multinucleated giant cells (MNGCs) aggregate in aged ovaries. Converse et al. demonstrated that MNGCs (presumed to arise from macrophage fusion) are exclusively present in aged ovarian tissue and co-localize with areas of immune infiltration. Laser capture microdissection revealed these cells highly express GPNMB, exhibit enrichment in oxidative phosphorylation and lipid metabolism pathways, and frequently cluster adjacent to CD4^+^ T cell aggregates (74). Additional studies have confirmed that MNGCs, unique to aging ovaries, also exhibit enriched gene expression patterns in proteolytic, lipid metabolism, and mitochondrial-related pathways (74).

In summary, combined results from human and mouse studies indicate that macrophage remodeling is a core feature of ovarian aging. However, the direction of polarization varies across different experimental contexts, suggesting that macrophage polarization does not represent a linear transformation but rather exhibits dynamic, stage-dependent alterations. Research evidence from middle-aged mouse models (10 months) indicates that at this stage, the ovaries are characterized by pro-inflammatory macrophage features. Specifically, this manifests as increased monocyte recruitment, inflammasome activation, and elevated levels of inflammatory mediators. These changes are closely associated with follicular atresia and endocrine function decline (29). In contrast, studies on late reproductive-stage mice (12–16 months) revealed that their ovarian microenvironment exhibited enhanced extracellular matrix remodeling and increased stromal fibrosis, along with enriched macrophage subpopulations associated with tissue repair and fibrotic responses (75). In these models, partial restoration of follicular integrity and hormonal balance can be achieved through drug intervention or regulation of macrophage subpopulations based on EVs, highlighting the functional relevance of macrophage plasticity.

Importantly, human single-cell sequencing and histological analysis provide complementary evidence for this stage-dependent remodeling. The age-related transition from tissue-resident macrophages to inflammatory monocyte-derived macrophages coupled with enhanced pyroptosis-associated signaling suggests that early inflammatory reprogramming is a conserved feature of ovarian aging. Furthermore, MNGCs specifically appear in aged non-human primate and mouse ovaries. They exhibit highly enriched oxidative phosphorylation and lipid metabolism pathways and frequently localize near areas of immune infiltration. The emergence of these multinucleated macrophage derivatives accompanied by increased collagen deposition and elevated stromal stiffness collectively supports the emergence of fibrosis-associated macrophage phenotypes in late ovarian aging.

Collectively, these cross-species findings support a stage-dependent model. Ovarian aging progresses from an early phase dominated by pro-inflammatory macrophages and characterized by chronic low-grade inflammation, to a late stage marked by reparative and fibrosis-associated macrophage phenotypes that mediate extracellular matrix deposition and stromal remodeling. This highly temporally ordered macrophage remodeling explains previously inconsistent findings across studies and highlights immune plasticity as the core mechanism linking chronic inflammation, tissue repair, and functional decline in the aging ovary.

### 3.2 POI

Due to difficulties in obtaining ovary tissue samples from patients with POI, most of evidence is from peripheral blood, follicular fluid, and animal models.

In peripheral blood immunophenotyping analysis, studies have demonstrated that patients with POI exhibit a reduced ratio of circulating CD14^+^ monocytes to NK cells, along with a relative enrichment of activated CD4^+^ T cells and plasma cells. Concurrently, elevated levels of C-reactive protein and specific cytokines are observed, consistent with the presence of chronic low-grade systemic inflammation in these patients (19). Early findings and subsequent validation studies indicate that while the total number of Tregs may show an upward trend in some POI populations, their effector Treg subpopulations exhibit functional defects (7, 76). Concurrently, researchers observed an expansion of CD4^+^CD69^+^ activated T cells, suggesting a qualitative remodeling between Tregs and effector T cells rather than a simple reduction in total Treg numbers. Cytokines, as signaling molecules of the immune system, exhibit complex expression patterns in POI. Nouri et al. reported elevated serum levels of IL-17, IL-21, and IL-23 in POI patients (77). Jiao et al. confirmed that IFN-γ and TNF-α synergistically induce granulosa cell apoptosis. Earlier studies also revealed decreased TGF-β1 levels in POI patients, accompanied by elevated IFN-γ (76). Collectively, these alterations indicate systemic dysregulation of the Treg/TH1/TH17 axis. At the functional level, Jiao et al. demonstrated that insufficient numbers of Tregs in POI, coupled with impaired suppressive function, lead to excessive Th1 responses. IFN-γ and TNF-α synergistically induce apoptosis in granulosa cells and inhibit steroid hormone synthesis; conversely, adoptive transfer of Tregs into mouse models partially rescues follicular loss and endocrine abnormalities. Another mechanistic animal study further supports this view: in a cyclophosphamide-induced POI model, dihydroberberine treatment restored Th17/Treg balance, reduced IL-17 and IL-6 levels, mitigated follicular loss, and improved estrous cycle regularity. This provides proof of concept that correcting immune imbalance can achieve ovarian function preservation (78). Notably, Th1/Th17 overactivation driven by Treg dysfunction not only directly damages ovarian cells but also promotes macrophage polarization toward the M1 phenotype through the secretion of cytokines such as IFN-γ and IL-17, thereby establishing a pathological network of interactions between lymphocytes and myeloid cells.

At the microenvironmental level of follicles, data from individuals with ovarian hyporesponsiveness and proteomics studies indicate that dysfunctional dominant follicles exhibit a trend toward increased monocyte and M1 macrophage characteristics. Single-cell RNA sequencing by Han et al. further revealed elevated proportions of inflammatory monocytes in follicular fluid from POI patients, alongside the loss of VEGFA-FLT1 signaling between monocytes and granulosa cells. Ligand-receptor interaction analysis indicated significantly enhanced proinflammatory signaling between monocytes and granulosa cells in POI (79). Additionally, Tang et al. (109) reported that follicular fluid from POI patients showed significantly elevated levels of IL-33 accompanied by reduced concentrations of its soluble decoy receptor sST2, and excessive IL-33 signaling may promote the recruitment and activation of monocytes/macrophages in the follicular microenvironment, thereby amplifying local inflammation. The polarization shift of macrophages from M2 to M1 not only directly impairs granulosa cell function but also recruits additional inflammatory cells through a positive feedback loop, establishing a self-sustaining inflammatory microenvironment (6).

In animal models, multiple toxin- or chemotherapy-induced POI models consistently demonstrated reduced M2 markers within ovarian tissue alongside increased M1 macrophage characteristics. In cyclophosphamide-induced POI models, Yang et al. observed significantly enhanced M1 polarization of ovarian macrophages and elevated inflammatory cytokine expression. Metformin intervention partially reversed these alterations, preserving granulosa cell survival and steroid synthesis function (80). Ren et al. further demonstrated that Caulerpin inhibits M1 polarization via the p53/NF-κB pathway, reducing ovarian levels of IL-1β, IL-6, and TNF-α, thereby protecting follicular architecture (81). These findings align with the core concept of ovarian macrophage biology, namely that M1 macrophages, which secrete large amounts of TNF-α, IL-1, and IL-6, exacerbate tissue damage, whereas M2 macrophages play a role in tissue repair.

In summary, immune alterations associated with POI share certain similarities with the inflammatory aging patterns observed in physiological ovarian aging, such as macrophage reprogramming and elevated SASP-like cytokines. However, immune abnormalities in POI emerge earlier and are more pronounced, potentially driven by diverse triggers including autoimmunity and exogenous injury (102).

## 4 Targeting immune microenvironment in ovarian aging and POI

**Figure 3:**
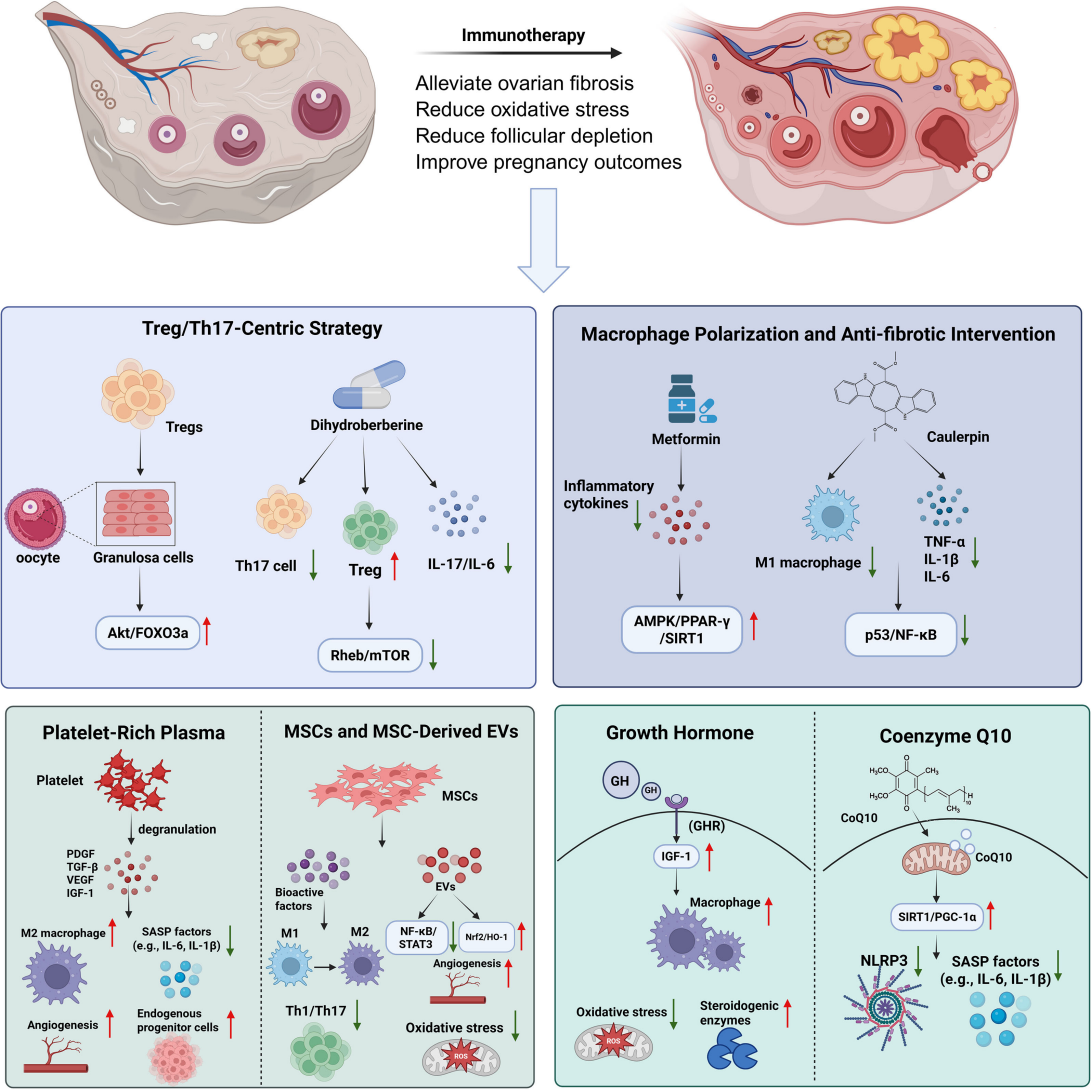
Multimodal therapeutic strategies for ovarian aging and POI. Therapeutic strategies for ovarian aging and POI include: (1) immune-centric approaches (Treg/Th17 balance, macrophage polarization) to mitigate inflammation and fibrosis; (2) systemic metabolic regulators (growth hormone, CoQ10) to reduce oxidative stress and senescence; (3) regenerative therapies (platelet-rich plasma, MSCs/EVs) to promote angiogenesis and tissue repair, collectively alleviating follicle depletion and improving clinical pregnancy rates.

POI and pathological ovarian aging represent complex processes involving the interplay of genetic, iatrogenic, and environmental factors with immune and metabolic dysregulation. Based on the core concept that chronic low-grade inflammation and immune-stromal interactions accelerate follicular depletion, multiple intervention strategies are currently employed to remodel the ovarian immune microenvironment and delay ovarian functional decline. These include systemic metabolic regulation, locally administered bioactive agents, and cellular or acellular regenerative therapies (**Figure 3**).

### 4.1 Treg- and Th17- targeted strategies

Given the dysregulation of the Treg/Th1/Th17 axis in POI, multiple research teams have attempted to directly restore the balance of these CD4^+^ T cell subsets. In a ZP3-induced POI mouse model, adoptive transfer of CD4^+^CD25^+^ Tregs partially restored AKT/FOXO3A signaling in granulosa cells, reduced follicular apoptosis, improved ovarian morphology, and elevated estradiol levels (82). Complementing cell therapies, researchers have also explored the regulatory effects of small-molecule drugs on Th17/Treg balance. Studies indicate that in cyclophosphamide (CTX) induced POI models, dihydroberberine inhibits the Rheb/mTOR signaling pathway, reduces Th17 cell proportion and serum IL-17/IL-6 levels, increases Treg proportion, ultimately alleviating follicular depletion and endocrine dysfunction (78). These findings confirm that targeting reprogrammed T cell subsets can protect ovarian structure and function; however, these strategies remain in the preclinical research phase.

### 4.2 Macrophage polarization and anti-fibrotic interventions

Beyond T-cell subset dysregulation, abnormal macrophage polarization and ovarian fibrosis represent another critical component of immune microenvironment impairment in POI. Chemotherapy-induced POI models consistently exhibit elevated proportions of pro-inflammatory M1 macrophages and worsened ovarian stromal fibrosis. Metformin, as a potential immunometabolic modulator, demonstrates protective effects in this context. Studies indicate that in CTX-induced POI mice, metformin activates the AMPK/PPAR-γ/SIRT1 axis, mitigates oxidative damage and inflammatory cytokine expression in granulosa cells, enhances follicle survival, and improves reproductive hormone levels (80). Recently, Nie et al. reported that metformin alleviates CTX-induced ovarian fibrosis by regulating the MIF/CD74 axis, reshaping macrophage-fibroblast communication, and reducing ovarian stromal collagen deposition (83). These findings indicate that metformin serves not only as a metabolic regulator but also as an intervention targeting the ovarian microenvironment and macrophages.

Other small molecules exhibit similar macrophage polarization regulation in POI models. For instance, caulerpin mitigates CTX-induced ovarian toxicity by suppressing p53/NF-κB-mediated M1 polarization, thereby reducing TNF-α, IL-1β, and IL-6 levels in mouse ovarian tissue (81). Coupled with the CD38/NAD^+^ data mentioned above (84, 85), these findings suggest that pharmacologically targeting key metabolic and inflammatory nodes such as AMPK, mTOR, CD38, and NF-κB can effectively restore ovarian immune microenvironment homeostasis.

### 4.3 Platelet-rich plasma

Regenerative medicine strategies can improve ovarian function by promoting tissue repair and remodeling the microenvironment, with PRP being a representative approach. PRP is rich in platelet-derived growth factors (PDGF, TGF-β, VEGF, IGF-1, etc.) and chemokines. Preclinical POI models demonstrate that intraovarian PRP injection reduces follicular atresia, alleviates inflammatory infiltration, increases the number of growing follicles, and simultaneously improves estrous cycles and hormone levels (86). Multiple small-sample studies and recent systematic reviews suggest that autologous intraovarian PRP injection may improve antral follicle count (AFC), oocyte retrieval rate, and embryo yield in patients with POR or POI. Some patients regain menstruation, with mild improvements in AMH and follicle-stimulating hormone (FSH) levels (87–90). Meta-analyses demonstrate that intraovarian PRP injection significantly increases AFC and the number of oocytes retrieved (both P<0.001). The pooled clinical pregnancy rate was 21% in women with poor ovarian reserve and 13.8% in those with POI. Clinical pregnancy rates showed a favorable trend, but statistical significance was not reported in the study (88). Mechanistically, in a ovarian ischemia-reperfusion injury model, PRP exerts protective effects by significantly decreasing tissue levels of the pro-inflammatory cytokine TNF-α, reducing mononuclear cell infiltration and vascular congestion in the ovarian stroma, and inhibiting activation of the NF-κB inflammatory signaling pathway in macrophages. These actions ameliorate the local ovarian inflammatory imbalance, thereby reshaping the ovarian immune microenvironment and attenuating ischemia-reperfusion-induced immune-mediated tissue damage (86, 91). However, due to inconsistencies in PRP preparation, dosage, and timing of intervention, coupled with the lack of large-scale randomized controlled trials, it currently remains an experimental treatment.

### 4.4 Mesenchymal stem cells and MSC-derived EVs

MSCs and their EVs represent one of the most extensively studied immunomodulatory and (86)regenerative therapeutic approaches in the field of POI. Systematic reviews and meta-analyses demonstrate that MSC therapy consistently improves ovarian weight, follicular count, estradiol levels, and reproductive outcomes while reducing granulosa cell apoptosis, oxidative stress, and inflammatory mediators such as TNF-α and IL-6 (92). Mechanistically, MSCs exert paracrine effects by secreting anti-inflammatory factors, growth factors, and EVs. This promotes macrophage polarization toward an M2-like phenotype, suppresses Th1/Th17 responses, and alleviates ovarian stromal fibrosis (92, 93).

MSC-derived exosomes (MSC-Exos), as a more controllable acellular therapeutic strategy, also demonstrate protective effects. Human umbilical cord MSC exosomes promote granulosa cell expansion and inhibit apoptosis via the Hippo pathway, improving ovarian function in chemotherapy-induced POI mice (94). Overexpression of miR-10a or IGF-1-carrying genetically engineered exosomes further enhances granulosa cell protection by mitigating oxidative stress and inflammatory damage (95, 96). MSC-Exos can reduce local ovarian inflammation by inhibiting the activation of the NLRP3 inflammasome pathway and reducing the release of pro-inflammatory cytokines such as IL-1β and IL-18. At the same time, they repair the morphology and structure of ovarian tissue, restore the integrity of ovarian microvasculature, and improve oxidative metabolic imbalance in the ovary, thereby reestablishing the metabolic homeostasis associated with ovarian hormone secretion and follicular development (97, 98). Current clinical studies remain in the early exploratory phase with small sample sizes. While short-term safety appears acceptable, long-term risks of tumorigenesis and ectopic tissue formation require rigorous monitoring (93, 99).

### 4.5 Growth hormone

Beyond immune regulation, metabolic and endocrine interventions also represent important avenues for improving ovarian reserve and responsiveness. Among these, GH is the most extensively studied adjunctive intervention. GH is commonly used in patients with diminished ovarian reserve (DOR) and poor ovarian response (POR), with its mechanisms of action overlapping to some extent with those in POI. Multiple randomized and non-randomized clinical trials, along with a recent network meta-analysis, demonstrate that GH supplementation during controlled ovarian stimulation improves oocyte retrieval rates, mature oocyte yields, and fertilization rates in POR/DOR populations. In certain protocols, it also enhances clinical pregnancy and live birth rates (99–101). Mechanistically, GH activates the IGF-1 signaling pathway in mouse models, induces double-strand DNA breaks in oocytes and granulosa cells, and increases the infiltration of ovarian macrophages, thereby upregulating local ovarian inflammatory responses. Concurrently, it modulates the number of granulosa cells to synergistically regulate the ovarian immune microenvironment. However, evidence for the direct immunomodulatory effects of GH in human POI remains insufficient (102). Therefore, current guidelines consider GH as an optional adjunctive therapy for some low-response or DOR patients. However, high-quality evidence in confirmed POI remains insufficient, precluding its classification as an independent immunotherapy (103).

### 4.6 Coenzyme Q10 and mitochondrial protection

Mitochondrial dysfunction and heightened oxidative stress are key hallmarks of ovarian aging. Coenzyme Q10, as a crucial mitochondrial antioxidant, has garnered significant attention in this field. CoQ10 serves as an essential component of the mitochondrial respiratory chain while exhibiting potent lipophilic antioxidant properties. In aged mouse models, CoQ10 supplementation restored mitochondrial membrane potential in oocytes, increased follicle numbers and ovulation rates, improved litter size, and reversed age-related oocyte dysfunction (104). Clinical studies indicate that 2–3 months of CoQ10 pretreatment (30–200 mg/day) in patients with DOR improves oocyte retrieval rates, number of MII-stage oocytes, high-quality embryo yield, and clinical pregnancy rates (105). Animal studies indicate that CoQ10 upregulates SIRT1/PGC-1α signaling, promotes mitochondrial biogenesis, and mitigates oxidative damage in granulosa cells and oocytes (106). CoQ10 may indirectly improve the ovarian immune microenvironment by reducing oxidative stress-associated SASP-like cytokines and inflammasome activation (107, 108); however, systematic evidence for its direct regulation of immune cell subsets in POI remains lacking.

In summary, intervention strategies for delaying ovarian aging and treating POI have evolved into a multi-tiered, multi-targeted system. Immunomodulatory approaches targeting Treg/Th17 imbalance and regulating macrophage polarization can directly correct disruptions in the ovarian immune microenvironment. Endocrine, mitochondrial, and antioxidant-focused therapies like growth hormone and coenzyme Q10 provide indirect protection by enhancing fundamental cellular functions; Regenerative approaches such as platelet-rich plasma, mesenchymal stem cells, and their exosomes delay follicular depletion and ovarian fibrosis by promoting angiogenesis, stromal remodeling, and anti-inflammatory repair.

However, current high-quality evidence primarily stems from preclinical studies or small-sample, highly heterogeneous clinical cohorts, with limited long-term safety data. Existing professional guidelines still classify these interventions—particularly platelet-rich plasma, mesenchymal stem cell-related therapies, and systemic immunosuppressive treatments—as experimental approaches. Future research should prioritize patient stratification based on pathogenesis, promote standardization of platelet-rich plasma and mesenchymal stem cell/exosome preparation protocols, conduct Phase I/II clinical trials targeting rationally selected immunometabolic endpoints such as ovarian reserve and live birth, and incorporate immune detection markers like single-cell sequencing and spatial omics into clinical studies to directly validate the reprogramming effects on the ovarian microenvironment.

## 5 Conclusions and future directions

In this review, we synthesized current evidence on how the ovarian immune microenvironment shapes ovarian development, supports cyclical remodeling, and contributes to pathological states such as POI and accelerated ovarian aging. From early folliculogenesis through ovulation and luteinization, immune cells and their mediators are not passive bystanders but integral components of the ovarian niche.

Across human and animal studies, several converging themes emerge. First, Ovarian aging and POI are both accompanied by a shift from a homeostatic to a pro-inflammatory immune state, characterized by altered macrophage polarization, accumulation of inflammatory monocytes, and T-cell imbalance. Second, these immune alterations are not restricted to peripheral blood. Single-cell and spatial analyses in follicular fluid and ovarian tissue reveal local enrichment of inflammatory myeloid clusters, activated inflammasome and complement signaling, and SASP-like cytokine profiles that interact with senescent stromal niches. Third, experimental models illustrate that modifying immune tone, whether by restoring Treg function, rebalancing Th17/Treg, or shifting macrophages away from a chronic M1 phenotype, can mitigate follicle loss, preserve steroidogenesis, and partially rescue ovarian function. Together, these data support a model in which immune–endocrine–metabolic crosstalk is a central determinant of ovarian health, and in which immune imbalance accelerates the transition from physiological aging to POI.

Looking ahead, several priorities can be delineated. At the mechanistic level, there is a need for deeper, multi-layered mapping of the ovarian immune landscape across the lifespan and disease states, integrating single-cell and spatial transcriptomics with proteomics, metabolomics, and immune receptor repertoire sequencing. Such efforts should explicitly link immune-cell states to functional readouts of follicle dynamics, oocyte competence, and stromal remodeling, and should dissect causality using targeted perturbations in refined animal models. Clinically, future studies should move beyond descriptive associations toward prospective, stratified trial designs. Patients with POI or accelerated ovarian aging could be subtyped according to immune signatures or genetic risk, and then enrolled into mechanism-driven interventional studies. A current limitation of research is the scarcity of patient samples, particularly ovarian tissue from women with POI. Moreover, most single-cell sequencing studies fail to provide detailed descriptions of the samples, such as whether the tissue was obtained from the ovarian cortex or medulla, how the tissue was collected, or the extent of extracellular matrix preservation. The lack of such details limits cross-study comparisons and meta-analyses, and makes it difficult to distinguish true biological variation from technical artifacts introduced by inconsistent sampling practices. In parallel, regulatory frameworks will need to keep pace with these advances, providing clear guidance on the development and monitoring of immunologically active therapies in reproductive-age women.

Ultimately, a more precise understanding of the ovarian immune microenvironment has the potential to transform both prognosis and therapy. Elucidating how it supports normal follicular development, how it gradually deteriorates with aging, and how it becomes pathologically dysregulated in POI will reveal novel intervention points. By coupling high-resolution immune profiling with rationally designed interventions and careful long-term follow-up, the field can move from broadly empiric approaches to truly personalized immuno-reproductive medicine, offering new avenues to preserve ovarian function, extend reproductive health span, and improve the quality of life for women at risk of POI and age-related decline.
